# Self-directed home-based neuromuscular electrical stimulation (NMES) in patients with advanced cancer and poor performance status: a feasibility study

**DOI:** 10.1007/s00520-020-05394-0

**Published:** 2020-03-16

**Authors:** Dominic O’Connor, Olive Lennon, Sarah Wright, Brian Caulfield

**Affiliations:** 1 School of Public Health, Physiotherapy and Sports Science, University College Dublin, Dublin, Ireland; 2 The Insight Centre for Data Analytics, O’Brien Centre for Science, University College Dublin, Dublin, Ireland; 3 Queens University, Belfast, Ireland; 4 Physiotherapy Department, Beacon Hospital, Dublin, Ireland

**Keywords:** NMES, Neuromuscular electrical stimulation, Advanced cancer, Rehabilitation, Sit-to-stand, Physical function

## Abstract

**Purpose:**

Concurrent neuromuscular electrical stimulation (NMES) involving sub-tetanic low frequency and tetanic high frequency which targets aerobic and muscular fitness is a potential alternative to conventional exercise in cancer rehabilitation. However, its safety and feasibility in patients with advanced cancer are unknown. The aim of this feasibility study was to determine safety and feasibility and evaluate changes in functional and health-related quality of life (HR-QoL) outcomes in individuals with advanced cancer and poor performance status after concurrent NMES. These results should help inform the design of future studies.

**Methods:**

Participants with advanced cancer and poor performance status (Eastern Cooperative Oncology Group scale ≥ 2) (*n* = 18) were recruited. The intervention included a novel NMES intervention implemented over a 4-week period. Functional exercise capacity, lower limb muscle endurance and HR-QoL were measured by 6-min walk test (6MWT), 30-s sit-to-stand (30STS) and European Organization for Research and Treatment quality of life questionnaire core-30 (EORTC QLQ C30) pre and post-intervention. Participants unable to complete the 6-min walk test completed the timed up and go test. Participant experience and the impact of the intervention on daily life were investigated through semi-structured interviews.

**Results:**

Ten of 18 participants completed the intervention. No adverse events were reported. Seven of 8 participants improved 6MWT performance (2 of 2 improved timed up and go), 8 of 10 participants improved 30STS and 8 of 10 participants improved Global quality of life. Perceived benefits included improved mobility and muscle strength.

**Conclusions:**

Neuromuscular electrical stimulation appears safe and feasible in advanced cancer and may improve physical and HR-QoL outcomes. Future prospective trials are warranted to confirm these findings prior to clinical implementation in an advanced cancer setting.

## Introduction

Neuromuscular electrical stimulation (NMES) offers an intuitively appealing alternative to conventional exercises such as aerobic and resistance exercise for clinical populations who find these exercises difficult. Muscle contractions can be achieved without voluntary input from the user (in either seated or supine positions for those with profound deconditioning), via electrical impulses delivered to motor nerves using surface electrodes placed over target muscle groups, typically using a handheld battery-powered stimulation unit [1]. Tetanic high frequency NMES (HF-NMES, > 20 Hz) increases muscle strength [2], whilst emerging evidence indicates that the application of sub-tetanic low frequency NMES (LF-NMES, 3–12 Hz) can augment aerobic energy metabolism and subsequently enhances cardiorespiratory fitness and exercise tolerance [3]. Therefore, a concurrent NMES exercise approach employing both LF and HF NMES exercise may help augment functional outcomes when appropriately prescribed in individuals unable to achieve therapeutic levels of voluntary exercise due to profound deconditioning.

Recent work has provided preliminary evidence of the safety and feasibility of adopting this novel concurrent NMES exercise approach in cancer survivorship. O’Connor et al. [4] evaluated the use of this approach (phase 1: LF-NMES, 4 Hz, continuous, 15–45 min; phase 2: HF-NMES, 20 Hz, 2–5 s on:10–15 s off, 15 mins, both phases progressed weekly) in a small group of adult cancer survivors with mixed cancer diagnoses who were undergoing or had recently completed treatment, were functionally independent but had been referred due to some restrictions in physically strenuous activity (Eastern Cooperative Oncology Group (ECOG) performance status 1 (ECOG 1)). The authors observed no adverse events and high adherence to the home-based protocol, with participants reporting that the technology was easy to use during their daily lives. In addition, they reported clinically meaningful improvements in physical function and health-related quality of life (HR-QoL) [4], with the greatest improvements observed in the more deconditioned patient. This finding is replicated in the NMES exercise literature in patients with chronic respiratory and critical illnesses [5, 6].

Many cancer survivors, particularly those with advanced disease require some assistance with daily activities and find voluntary exercise unachievable due to profound deconditioning and/or other complications [7] highlighting the need for alternative interventions. Impaired functional ability is observed in 60–70% of advanced patients and is associated with increased morbidity, loss of independence and significantly diminished HR-QoL [8]. It can be argued that advanced cancer patients have the greatest need for supportive interventions; however, this population is underrepresented in clinical research [9]. Interestingly, early work suggests that exercise preferences in people with advanced cancer may favour unsupervised, home-based NMES exercise over voluntary exercise programmes [10]. To date, no studies have reported on a concurrent NMES exercise intervention in patients with advanced cancer and profound deconditioning.

This feasibility study aimed to expand on the findings of O’Connor et al. [4] and provide early explorative data on the safety, feasibility and effects of a personalised and progressive concurrent NMES exercise programme in patients with advanced cancer and poor performance status as rated by the ECOG scale.

## Materials and methods

### Design and study participants

This single-site, feasibility study followed study participants for a period of 4 weeks, with two measurement time points. Measurements of functional capacity and participant reported outcomes (PROs) were recorded at baseline and post NMES exercise intervention. Participants’ experiences of concurrent NMES exercise were recorded after the intervention.

Between August and December 2018, oncology patients who had been admitted to an oncology inpatient ward or were attending an oncology day unit at a large private teaching hospital (Beacon Hospital, Dublin) were invited to take part by their treating physician. Inclusion criteria were the following: adults with advanced disease (stage IV), an ECOG score of ≥ 2 (ambulatory and capable of all self-care but unable to carry out any work activities; up and about more than 50% of waking hours), and deemed inappropriate (due to impaired functional capabilities) for inclusion in a group-based oncology exercise programme (Fit for Life), available as part of a clinical service for oncology patients. Exclusion criteria were serious cardiac arrhythmias, cardiac pacemaker, any cognitive impairment which may affect their ability to apply NMES safely unsupervised, deep vein thrombosis within the previous 6 months and metastatic lesions to the femur. Participants were fully informed of all experimental procedures prior to giving written informed consent. This study was approved by the Beacon Hospital and University College Dublin ethics committees.

### NMES intervention

The NMES exercise intervention has previously been described in detail [4]. In brief, the 4-week concurrent NMES exercise intervention was delivered using a handheld muscle stimulation unit (INKO RS, Bio-Medical Research Ltd., Galway, Ireland) and four adhesive gel electrodes (17 × 10.3 cm) placed on each leg (× 2 proximal and distal quadriceps, × 2 proximal and distal hamstrings) and applied via a pair of neoprene garments which were secured by velcro straps. The participants trained unsupervised at home using a standard weekly progressive prescription (14 sessions; Table 1) which was personalised weekly (session frequency and duration) and delivered LF and HF-NMES phases during each session.

**Table 1:** Standard prescription and progression guideline

Time	Phase	Standard progression (duration/on:off)	Session frequency (no./week)
Week 1	LF-NMES	3 × 3 min ramp	2
	HF-NMES	N/A	2
Week 2	LF-NMES	3 × 5 min ramp*	3
	HF-NMES	2 s on:15 s off	3
Week 3	LF-NMES	25 min continuous	4
	HF-NMES	5 s on:15 s off	4
Week 4	LF-NMES	30 min continuous	5
	HF-NMES	5 s on:10 s off	5

#### Personalised and progressive NMES

**Fig. 1:**
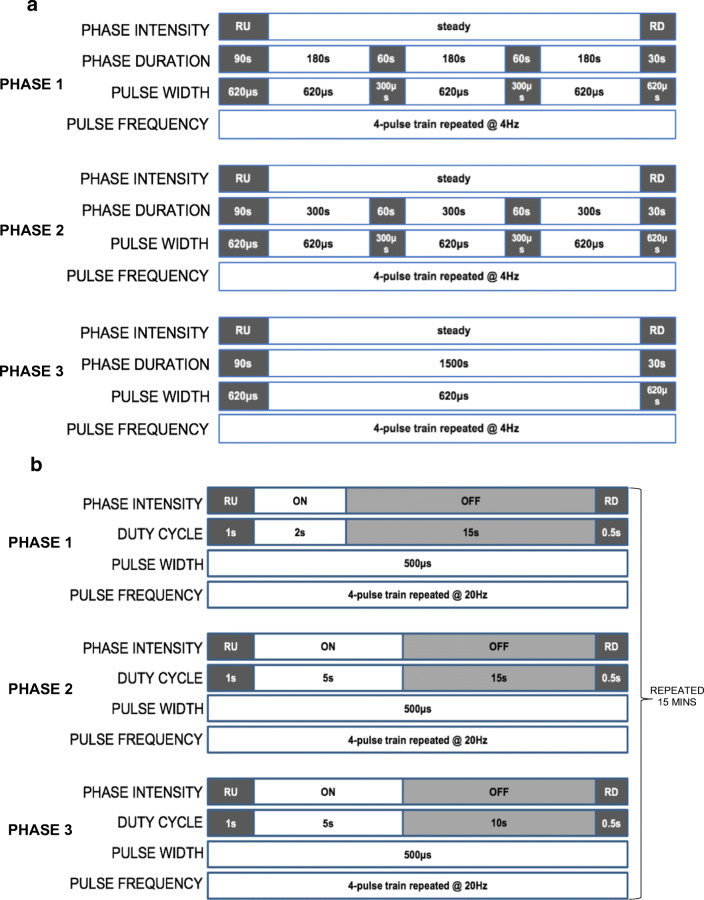
Schematic representation of LF-NMES intermittent delivery (a) and HF-NMES duty cycle (b) progressions

As tolerability is a major determinant of the response to NMES [11], a progressive and personalised prescription was developed. A novel intermittent delivery of the LF-NMES programme (Fig. 1a) was used in week 1 (phase 1) with reduction of the pulse width from 620 to 300 μs used as the means of introducing relative “rest” periods to the intermittent programme to accommodate habituation for unaccustomed users. However, in contrast to the previous protocol [4], patients were progressed directly to continuous delivery if deemed appropriate, as identified during a 10-stage incremental NMES protocol session (could tolerate current intensities beyond 15-min, i.e. ≥ 70 mA). The 10-stage incremental NMES protocol involved the stimulation intensity being increased by the study participant every 3 min in equal increments of 14 mA (10% of maximum current output; 140 mA) from a starting point of 14 mA. If participants, at the start of a new stage, could not tolerate an increase of + 10%, the maximum tolerable increase was achieved prior to termination of the session at the end of that stage. This session (completed in the hospital) also acted as a familiarisation session whereby the safe and correct use of the unit was demonstrated by the study investigator. Participants were provided written instructions on the safe use of the NMES units which could be referred to when at home.

Progression in the home-based LF-NMES intervention involved increased weekly session duration (5–10 min per week). In the HF-NMES protocol, the duty cycle (on:off cycle) increased weekly from 2 s:15 s to 5 s:15 s to 5 s:10 s and constant thereafter as previously reported (Fig. 1b) [4]. Session frequency progressed weekly from 2x/week in week 1 to 5x/week in week 4 (Table 1). Participants were provided diaries to record session self-reported compliance (session duration and intensity). To maximise the self-reported compliance with the intervention, weekly phone calls were completed to identify and solve problems and prompt continued increase in stimulation intensities as tolerated.

### Outcome measures

Safety and feasibility were assessed following the intervention via reported adverse events and, participant recruitment and retention, and self-reported compliance with the intervention prescription. To assess the concurrent NMES protocol, objective tests of physical function and psychosocial outcomes were assessed before and after the intervention. Physical assessments included functional muscle strength and aerobic exercise capacity. Participant reported outcomes measured HR-QoL. Participant experience was determined following the intervention using semi-structured interviews.

### Physical assessment

#### Functional muscle endurance

Lower limb muscle endurance was assessed using the 30-s sit-to-stand (30STS). The 30STS required patients to stand up from, and sit down on a 45 cm padded chair with no armrests as many times as possible in 30 s [12]. Participants could use their hands to help them stand if required and were provided standardised verbal encouragement to continue to sit and stand throughout the test. Participants completed one trial. A change in 30STS score of ≥ 2 reps was considered the minimal clinically important difference (MCID) [13].

### Functional exercise capacity

#### Six-minute walk test

Functional exercise capacity was assessed using a six-minute walk test (6MWT) which is a simple walking test, often used as a surrogate measure of aerobic capacity. Participants were instructed to walk as far as possible in the 6-min, back and forth along a 20-m corridor, turning briskly around the markers at each end. Participants could slow down, stop and rest if necessary. Standardised moderate verbal encouragement was provided every 2 min by the same investigator to each patient. The distance walked in 6 min was recorded to the nearest meter. An improvement in distance of 30.5 m was considered the MCID [14].

#### Alternate functional test

##### Timed up and go test:

Functional exercise capacity was assessed using the timed up and go test (TUG). Patients were required to stand up from a 45 cm chair, walk 3 m, turn around, walk 3 m back and sit down again, walking at a preferred pace. The use of a walking aid was allowed. The test was completed twice, with the mean score being recorded. A change of 3 s in TUG time was considered the MCID (size of the relative change; 23%, mean TUG score: 14.3 s, (14.3 × 0.23 = 3.2)) [15].

### Participant reported outcomes

#### HR-QoL

The multidimensional European Organisation for the Research and Treatment of Cancer Quality of Life Questionnaire Core 30 (EORTC QLQ-C30) was used to assess HR-QoL. The EORTC QOL-C30 contains five functional scales and a global health status/quality of life scale. Functional scales assess physical function, role function, emotional function, social function and cognitive function. The global health status/QoL scale rates overall health and quality of life. Using the EORTC scoring manual, a linear transformation was used to standardise the raw score, so that scores ranged from 0 to 100. A higher score represents a higher level of global QoL and functioning. A change in subscale score of 5–10 was considered the MCID [16].

#### Participant experience

Participants were invited to complete semi-structured interviews to explore their experiences of using the NMES units and garments during the intervention, and its impact on their daily lives. Interviews were carried out by the principal investigator after the post-intervention assessments. Questions were open-ended and were tape-recorded. Recordings were transcribed verbatim.

### Data management and analysis

Continuous data are expressed as mean (SD). Data analysis for safety and feasibility objectives of this study was descriptive. For functional outcomes, paired sample *t* tests were carried out to test for differences between time points. For HR-QoL data, median and interquartile range were reported, and differences were examined using a Wilcoxon signed rank test. Responses to interview questions were analysed using inductive content analysis and open coding to explore participants’ experience of the intervention. Interviews were independently coded by one researcher.

## Results

### Recruitment

Twenty-six participants were identified and invited to take part in the study. Eighteen (14 females, 4 males) provided written informed consent. Reasons for not consenting included lack of interest (*n* = 4) and health deterioration prior to giving informed consent (*n* = 4). Twelve participants completed the baseline assessments, and 10 completed the study (Table 2). Reasons for withdrawal prior to baseline assessment were medical complications (*n* = 6). Two of the 12 participants who completed baseline assessment withdrew from the study due to reasons not associated with the intervention, namely, medical complications (*n* = 1) and death (*n* = 1). Baseline assessments were scheduled within 24 h of providing informed consent for in-patients within the ward and the next available day for those attending the day clinic. Participant characteristics are presented in Table 2.

**Table 2:** Participant characteristics of those who completed the study

	Mean (SD)
Age (years)	60 (9)
Range	50–75
Weight (kg)	73 (20)
BMI (kg/m^2^)	26.0 (5.8)
	N (%)
Sex	
Male	3 (30)
Female	7 (70)
Married	
Y	9 (90)
N	1 (10)
ECOG	
2	8 (80)
3	2 (20)
Cancer diagnosis	
Colorectal	4 (40)
Pancreatic	2 (20)
GBM	1 (10)
Lung	1 (10)
Gastric	1 (10)
Ovarian	1 (10)
Treatment	
Surgery	3 (30)
Chemotherapy	10 (100)
Radiotherapy	2 (20)
Immunotherapy	1 (10)
Comorbidities	
Hypertension	4 (40)
CIPN	2 (20)
Anxiety	1 (10)
Type 2 diabetes	1 (10)
Asthma	1 (10)
Impaired vision	1 (10)

*BMI* body mass index, *CIPN* chemotherapy induced peripheral neuropathy, *ECOG* Eastern Cooperatove Oncology Group, *GBM* glioblastoma multiforme

### Safety

No serious adverse events were reported. Non-serious adverse events were reported in one participant and involved a decreased tolerance to current intensities in the days immediately following chemotherapy. Due to their baseline functional disability, the 6MWT was not feasible for two participants, therefore were assessed for functional mobility by TUG.

### Adherence

The mean number of NMES sessions completed across the 4-week intervention was 12 (3). Five participants completed the NMES exercise intervention with 100% adherence (14/14). The primary reason for missed sessions was exacerbation of disease-related symptoms and included chronic fatigue, nausea and illness. The initial to final mean NMES exercise intensities reported by participants were 65.4 (16.7) mA to 85.7 (17.5) mA for the LF-NMES phase and 59.5 (17.1) mA to 79.0 (15.2) mA for the HF-NMES phase.

### Physical function

Functional muscle endurance significantly improved on average by 3 (4) repetitions from 7 (3) repetitions to 10 (3) repetitions (*p* = 0.03). Improvements which exceeded the MCID were observed in 6 of the 10 participants. Two participants demonstrated a deterioration in STS performance of 1 and 2 repetitions respectively.

Functional exercise capacity improved on average by 77 (86) m from 232 (69) m to 309 (61) m (*p* = 0.04). Improvements which exceeded the MCID were observed in 7 of the 8 participants who were suitable for this assessment. In one participant, the distance decreased by 100 m (− 32%). Two participants were deemed unsuitable for assessment via 6MWT, so were assessed for functional mobility via TUG. Both participants improved their TUG performance by 24% and 79% respectively, exceeding the MCID threshold, with a mean improvement of 22.7 (23.2) seconds. Both participants required the use of a walking aid to complete the test at baseline. At post-testing, one participant completed the test unaided (Table 3).

**Table 3:** Physical performance assessment results at baseline and post NMES intervention

	Baseline	Post	*p* value
30 s sit-to-stand (reps)	7 (3)	10 (3)	0.026
6-min walk test (m)	232 (69)	309 (61)	0.040
Timed up and go (s)*	37.6 (17.1)	14.9 (6.1)	0.399

*N = 2

### Health-related quality of life

Global QoL significantly improved across the intervention period from a median (IQR) point score of 29 (25–50) to 67 (38–67). Only one functional scale, role, significantly improved across the intervention period from a median point score of 0 (0–29) to 58 (38–67). No other significant changes were observed (Table 4).

**Table 4:** Global health QoL and functional scales

	Baseline	Post	p value
Global QoL	29 (25–50)	67 (38–67)	*p* = 0.031
Physical	70 (38–90)	63 (55–72)	*p* = 0.725
Role	0 (0–29)	58 (38–67)	*p* = 0.020
Emotional	71 (58–100)	79 (69–98)	*p* = 0.500
Cognitive	67 (33–83)	83 (67–100)	*p* = 0.235
Social	25 (4–50)	50 (38–67)	*p* = 0.092

### Participant experience

Seven participants completed qualitative interviews. Example codes and illustrative quotes from the qualitative interviews can be viewed in Table 5. *Positive Aspects:* all participant reported subjective improvements in lower limb strength. Two participants reported increased mobility. *Negative Aspects:* all participants reported some difficulty applying and removing the garments. Three of these participants reported that help (spouse or family member) was required to apply and remove garments across the intervention period. No other negative experiences were reported.

**Table 5:** Example codes derived from inductive content analysis and representative quotes

Perceived improvements in strength	
“I feel more mobile now which I think is probably due to increased strength in my legs”	
“I feel like my leg strength has improved massively since my surgery”	
“I feel like I gained some strength in my legs, but I did not use the unit much so it’s hard to tell	
“My legs felt stronger when getting out of bed, not as wobbly as before”	
“I feel like my leg strength improved and was noticeable after around 10 days…I feel my legs are stronger and my mobility is easier, so it was well worth using”	
NMES garment application difficulties	
“It can be a bit fiddly to put on and take off until you get used to it, but after that it is fine and quick”	
“The first time putting on was a bit fiddly but we got the hang of it after the first few sessions	
“Putting on the garments was a bit of a hassle to start”	
“It was hard to put on at the start but did not take long after the first 2 sessions”	

## Discussion

This is the first study to report on the safety and feasibility of concurrent NMES exercise in individuals with advanced cancer and poor performance status. Our results suggest that this intervention is safe and feasible for those unable to attend conventional exercise programmes due to profound deconditioning. No serious adverse events were reported, which are in agreement with previous studies in cancer rehabilitation [4, 17]. Of the ten participants who completed the intervention, a mean adherence of 12 sessions was recorded across the 4-week time period, with five participants achieving 100% adherence. Four of the remaining 5 participants exceeded 70% adherence. Therefore, despite the nature of advanced cancer, strong adherence to the NMES intervention was observed. In addition, a pattern of improvement was observed across measures of functional endurance, exercise capacity and HR-QoL. The results presented in this study provide preliminary evidence for use of a concurrent NMES exercise programme as a supportive intervention in adults with advanced cancer and poor performance status.

A strength of this study is the inclusion of a study population largely underrepresented in clinical research [9]. Individuals with advanced disease are likely to benefit most from supportive interventions due to higher symptom burden and functional impairment. Adherence to the NMES exercise intervention in this study population was high following commencement of the protocol (> 70%). However, participants who withdrew from the study did so due to medical complications not associated with NMES exercise, and one participant unfortunately died due to disease progression. This highlights the unstable nature of advanced cancer and the challenges faced by practitioners attempting to implement NMES exercise in this setting. Indeed, high attrition rates (~ 50%) are commonly reported in studies in advanced cancer lasting more than a few weeks, and this must be considered during study design/power calculations [18]. This study provides useful data on recruitment and attrition for those planning future, large controlled trials in this population.

The main limitation of this exploratory study is the small sample of participants. In the current study, the majority of cases showed improvements in functional muscle strength (8/10), aerobic exercise capacity (7/8) and functional mobility (2/2) at a time when functional decline might otherwise have been expected. Of the 8 participants who improved functional muscle endurance, 6 exceeded the MCID threshold [13], whilst all participants who improved 6MWT distance (*n* = 7) and both participants who improved TUG exceeded the MCID threshold. However, despite these findings, causality cannot be inferred from an uncontrolled observation, which may occur by chance [19]. That said, the high number of responders suggests promise for interventions of this nature, highlighting the need for further controlled studies to expand on these findings.

An important methodological observation from this study must be highlighted. Whilst results from this study compare favourably with those of our previous work [4], the major difference is that participants differed greatly in their performance status (ECOG 2 vs ECOG 1—restricted in physically strenuous activity but ambulatory and able to carry out work of a light or sedentary nature). As such, participant feedback regarding the wearable NMES garments also differed significantly between studies. In the current study, hitherto unreported difficulties applying NMES garments independently were identified, highlighting different experiences in those with advanced cancer and compromised functional abilities during NMES exercise application. These difficulties may present a barrier to long-term adherence in this group. However, the inclusion of electrodes within wearable garments allows the user to accurately apply all electrodes simultaneously, with consistent application likely leading to more effective muscle contractions. Therefore, in individuals with advanced disease and physical limitations inhibiting independent application, the importance of an existing and supportive social network in home NMES application is underscored [20]. Future trials should consider family/carer support in the intervention process.

To summarise, the results of this study suggest that concurrent NMES exercise is safe and feasible in adults with advanced cancer and poor performance status. Importantly, NMES exercise may be an effective supportive intervention to help improve functional outcomes during a time when a deterioration in physical function might otherwise have been expected. Future large controlled trials are warranted to confirm these preliminary findings prior to clinical implementation of this technology in this setting.
